# Level of Necrosis in Feline Mammary Tumors: How to Quantify, Why and for What Purpose?

**DOI:** 10.3390/ani14223280

**Published:** 2024-11-14

**Authors:** Joana Rodrigues-Jesus, Ana Canadas-Sousa, Marta Santos, Pedro Oliveira, Ana Catarina Figueira, Carla Marrinhas, Gonçalo N. Petrucci, Hugo Gregório, Flora Tinoco, Andrea Goulart, Helena Felga, Hugo Vilhena, Patrícia Dias-Pereira

**Affiliations:** 1 Department of Pathology and Molecular Immunology, School of Medicine and Biomedical Sciences, ICBAS-UP, University of Porto, 4050-313 Porto, Portugal; jjoanarodrigues@gmail.com (J.R.-J.); canadas.ana@gmail.com (A.C.-S.); 2 Centre for Investigation Vasco da Gama (CIVG), Department of Veterinary Sciences, Vasco da Gama University School, 3020-210 Coimbra, Portugal; acfigueira@gmail.com (A.C.F.); carlamarrinhas@hotmail.com (C.M.); hcrvilhena@hotmail.com (H.V.); 3 Department of Microscopy, School of Medicine and Biomedical Sciences, ICBAS-UP, University of Porto, 4050-313 Porto, Portugal; mssantos@icbas.up.pt; 4 Department of Populations Studies, School of Medicine and Biomedical Sciences, ICBAS-UP, University of Porto, 4050-313 Porto, Portugal; pnoliveira@icbas.up.pt; 5 OneVet Veterinary University Hospital of Coimbra (HVUC), 3020-210 Coimbra, Portugal; 6 OneVet Veterinary Hospital of Baixo Vouga (HVBV), 3750-742 Águeda, Portugal; 7 OneVet Veterinary Hospital of Porto (HVP), 4250-475 Porto, Portugal; goncalopetrucci@gmail.com; 8 Department of Animal and Veterinary Sciences, University Institute for Health Sciences, CESPU, CRL, 4585-116 Gandra, Portugal; hugogregvet@hotmail.com; 9 Animal and Veterinary Research Centre (CECAV), University of Trás-os-Montes e Alto Douro, 5000-801 Vila Real, Portugal; 10 AniCura Veterinary Hospital Centre (CHV), 4100-320 Porto, Portugal; 11 Dra. Flora Tinoco Veterinary Clinic, 4475-498 Maia, Portugal; cvfloratinoco@gmail.com; 12 Oeste Veterinary Centre, 2410-134 Leiria, Portugal; centroveterinariodooeste@gmail.com; 13 Clínica dos Gatos Veterinary Clinic, 4100-207 Porto, Portugal; lenafelga@gmail.com; 14 Associate Laboratory of Animal and Veterinary Sciences AL4AnimaLS, 1300-477 Lisbon, Portugal

**Keywords:** feline mammary tumors, interobserver agreement, prognosis, stereology, tumor necrosis

## Abstract

Necrosis is a common finding in human and animal neoplasms. The percentage of tumor necrosis is included in tumor grading schemes in veterinary oncology; however, evaluation methods are often overlooked. Different studies have assessed the prognostic value of tumor necrosis in feline mammary tumors with contradictory results, which could be related to methodologic variability. In this study, a comprehensive evaluation of tumor necrosis in feline mammary tumors (FMTs) was conducted, by applying a semi-quantitative and a quantitative methodology for assessing necrosis. The interobserver agreement, the relationship with clinicopathological characteristics and the prognostic value of necrosis were analyzed in 154 FMT cases. Although subjectivity in the assessment of necrosis existed, an almost perfect agreement (weighted quadratic k = 0.851) between two observers was obtained. Furthermore, there was a significant positive correlation between the semi-quantitative and quantitative methods. Necrosis was more common and more extensive in malignant tumors than in their benign counterparts. Despite the non-significant results in the survival analysis, extensive necrosis was significantly associated with aggressive clinicopathological features, such as higher histological grade, high mitotic count and lymphovascular invasion. Our results support the potential relevance of necrosis in FMT.

## Simple Summary

Tumor necrosis can be frequently found in malignant tumors and its prognostic value has been confirmed in multiple solid tumors in human and veterinary medicine. We evaluated the level of necrosis in 154 cases of feline mammary tumors (FMTs) using semi-quantitative and quantitative methods and found that tumor necrosis is particularly common in malignant tumors with clinicopathological characteristics associated with aggressive biological behavior. In addition, there was a positive correlation between the semi-quantitative and the quantitative methods, and the former had a high interobserver agreement. These results provide evidence of the importance of necrosis in FMTs and support the implementation of a semi-quantitative evaluation of necrosis in the FMT diagnostic routine.

## 1. Introduction

Necrosis, otherwise known as uncontrolled cell death, typically involves groups of cells and occurs following external irreversible cell damage. This results in cell swelling (oncosis) with rupture of the cell membrane, release of cellular contents and a consequent inflammatory reaction [1,2]. During tumor development, there is often insufficient blood supply, leading to a hypoxic and ischemic microenvironment, which ultimately results in necrosis [3,4]. Furthermore, the hypoxic milieu, together with the increased metabolic rate of neoplastic cells and the inflammatory response generated by necrosis, contribute to the overproduction of free radicals such as reactive oxygen species (ROS). This leads to oxidative stress, promoting further DNA damage and mutagenesis, thus contributing to tumor progression and metastasis [5,6,7].

In veterinary medicine, the percentage of tumor necrosis is currently used in tumor grading schemes of some malignant tumors, such as canine soft tissue sarcomas, canine splenic hemangiosarcoma, canine pulmonary carcinoma and feline and canine osteosarcoma [8]. It is presumed that only histological assessment is considered; however, there is a general lack of clear definition regarding the methods employed to assess necrosis, and whether an estimation of the extent of necrosis was performed during the macroscopic examination and/or histological evaluation. Furthermore, due to variations in the trimming technique and the fact that inclusion of macroscopically necrotic areas is often avoided, the histological extent of necrosis might not be an accurate representation of the whole tumor, hence introducing a bias during its microscopic assessment [8,9].

Studies in human solid tumors, including breast cancer, have demonstrated the prognostic value of tumor necrosis in such neoplasms, with necrosis being associated with clinicopathological features of a worse prognosis [10]. Tumor necrosis in feline mammary carcinoma (FMC) has been evaluated in a qualitative or semi-quantitative manner, being classified as absent or present, or categorized according to its extent; however, most methods are ill-defined [11,12,13,14,15]. Nonetheless, necrosis is a frequent finding in FMC, being present in 55.6–89.8% of the cases reported in the literature [11,12,14,16,17]. In the current official classification system for mammary tumors in domestic animals, it is emphasized that the haphazardly distributed necrotic foci, contrary to ischemic central necrosis, should be considered a reliable criterion of malignancy [18]. The latter form of necrosis can also be observed in large benign lesions [18]. Even though necrosis assessment methodology is not specified in most of the published studies of FMTs, it is plausible that overall necrosis, irrespective of its type and its pattern, was considered [11,12,13,14,15]. Furthermore, the prognostic value of tumor necrosis has yielded conflicting results in survival studies of FMC, which could be related to different definitions and variation on the methodological assessment of this morphological feature [11,12,13,15,19]. It is also important to stress that when morphological parameters are semi-quantitively assessed for grading mammary tumors, the interobserver agreement is low to moderate [20,21]. Stereology has been applied in the context of veterinary oncology as an objective, unbiased tool to perform reproducible estimations of morphological parameters [22,23,24]. In FMTs, to our best knowledge, the interobserver agreement in necrosis evaluation is unknown, and objective, unbiased approaches, as those using a stereological tool, have never been used to quantify necrosis.

Thus, the aims of this study included the following: (a) the evaluation of the presence of various types and patterns of histological necrosis (i.e., overall, ischemic central, random and comedo pattern) in a case series of queens with benign and malignant mammary neoplasms; (b) the application of a semi-quantitative and a quantitative stereological method for the assessment of overall necrosis in benign and malignant tumors; (c) analysis of the interobserver agreement in the semi-quantitative evaluation in benign and malignant neoplasms; (d) a comparison of the semi-quantitative and the quantitative necrosis levels in benign and malignant tumors; (e) analysis of the relationship between necrosis (i.e., presence/absence of central ischemic, random and comedo necrosis, semi-quantitative and quantitative level of necrosis) and clinicopathological features in the malignant tumors; and (f) assessment of the prognostic value of necrosis on the disease-free interval (DFI), overall survival (OS) and tumor-specific survival (TSS) of queens with malignant tumors.

## 2. Materials and Methods

The present study included 154 female cats diagnosed with 231 spontaneous benign and malignant mammary tumors, in the Laboratory of Veterinary Pathology (LabPatVet) of the School of Medicine and Biomedical Sciences, University of Porto (ICBAS-UP) between 2010 and 2023. None of the animals included in the study had previous history of malignant mammary neoplasms, and in cases with disease recurrence only the first diagnosis was considered.

Mammary samples were fixed in 10% neutral buffered formalin and routinely processed into paraffin blocks, cut into 3 µm sections and stained with hematoxylin and eosin (HE). One to two representative cross-sections of each tumor were embedded for histopathological diagnosis and, when identified, regional lymph nodes were also routinely processed. Recorded clinicopathological data included age, type of tumor growth, ulceration, tumor size (larger axis, post-fixation), clinical stage, histological classification, histological grade and respective individual criteria, perilesional inflammatory infiltrate, lymphovascular invasion, lymph node status and distant metastasis status. Histological classification and histological grading (both Elston and Ellis (EE) [25] and Mills and co-workers (MGS) [13]) were performed according to the Surgical Pathology of Tumors of Domestic Animals pertaining to the mammary tumors [18]. Assessment of peritumoral inflammatory reaction was conducted as described previously [26] and clinical staging was attributed according to the modified World Health Organization criteria [27].

The follow-up data were retrieved from the medical records of the animals included in the study. Overall survival was defined as the time interval between diagnosis (considered as the day of surgery) and death by any cause. Tumor-specific survival was defined as the time period between diagnosis and death or euthanasia due to disease progression and/or loss of quality of life due to neoplastic mammary disease. Disease-free interval was defined as the time interval between excision of all detected tumors and disease progression (mammary and/or regional lymph node recurrence or development of distant metastasis). Inclusion criteria considered for the survival analysis comprised the following: (a) 2 years of follow-up, (b) no history of other malignant non-mammary tumors, and (c) surgery as the only treatment with a curative intent. The presence of overall, random, ischemic central and comedo necrosis, as well as the level of necrosis on the semi-quantitative and stereological quantitative assessments, was analyzed regarding the aforementioned endpoints.

**Figure 1:**
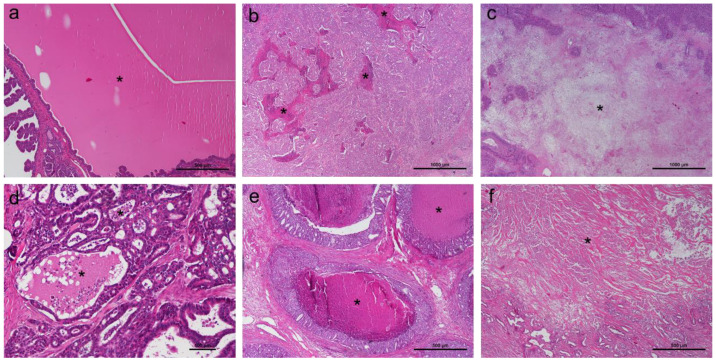
(**a**) Mammary secretion (*), HE, 4×. (**b**) Random necrosis (*), HE, 2×. (**c**) Ischemic central necrosis (*), HE, 2×. (**d**) Mammary secretion with inflammatory cells (*), HE, 10×. (**e**) Comedo necrosis (*), HE, 4×. (**f**) Ischemic central necrosis (*), HE, 4×.

Every neoplasm was first evaluated regarding the presence or absence of overall histological necrosis, ischemic central necrosis, random necrosis and comedo necrosis (Figure 1). Areas presenting loss of staining affinity with sheets of lightly eosinophilic material, ghost cells, nuclear pyknosis, karyorrhexis and/or karyolysis and cytoplasmic debris, as well as areas of amorphous, clustered brightly eosinophilic material often containing basophilic nuclear debris were considered necrotic tissue [2,9]. Ischemic central necrosis was considered when the tumor displayed a central, usually large, area of necrotic tissue, whereas haphazardly distributed foci of necrosis were included under random necrosis. Comedo necrosis was defined as round areas of necrosis bordered by neoplastic cells [18].

A semi-quantitative assessment of the extension of overall necrosis in a whole cross-section (selected based on the cross-section with the best quality and larger tumoral area; each case ranging from one to six slides, depending on the tumor’s size) of each tumor was performed by two independent observers (PDP and JRJ). This was performed by scanning every slide of the tumor section at lower magnification (4× and 10×), further amplifying when confirmation of dubious or inconspicuous necrotic areas was necessary and assigning one of the five following categories: absence of necrosis (score 0), <10% (score 1), 10–24% (score 2), 25–49% (score 3) or ≥50% (score 4).

**Figure 2:**
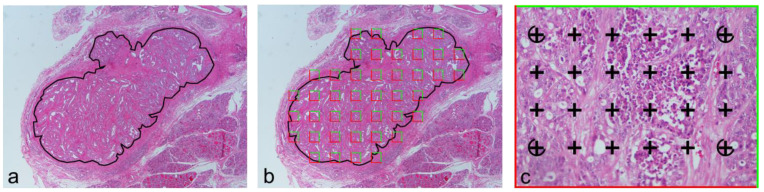
Representation of stereological estimation of relative volume (V_v_) of the necrosis within tumor parenchyma using the point-counting method. (**a**) Firstly, the entire cross-section of the tumor is manually delineated at low magnification. (**b**) Then, the software automatically selects 20 random and systematically sampled fields within previously delineated tumor area. (**c**) A frame with a point grid superimposed in each field of view; points lying on necrosis are counted; encircled points are counted to define the reference space.

A quantitative assessment of necrosis was also performed by one observer (JRJ) using a stereological method (Figure 2), blinded to the previous assessment results. This was achieved by using the software CAST-Grid (Version 1.5, Olympus, Albertslund, Denmark), which controlled a workstation encompassing a microscope (Olympus, BX50, Tokyo, Japan), a charged-coupled device (CCD) video camera (Sony, Tokyo, Japan) connected to a computer monitor and a motorized stage (Prior, Cambridge, United Kingdom) for xy precise motion. The stereological methodology was the point-counting method and consisted of the counting of points lying on necrotic material (object of interest) and points lying on tumor parenchyma (reference space) [23,28]. Points of interest were considered those hitting areas with loss of differential staining, comprising homogenous, mostly amorphous, eosinophilic material and/or degenerating and karyorrhectic debris [2,9]. Points hitting hemorrhage, ulceration, hyalinization, mammary gland secretion, mucin and blank spaces were not considered points of interest, even when these were within a necrotic area. Moreover, only points inside the tumoral area were considered; therefore, any point lying on the tumor capsule or the peritumoral area was omitted from the counting. Each tumor section was outlined using the CAST-Grid software, after which 20 fields (20× magnification) per slide were analyzed using a systematic sampling approach. The relative volume (V_v_) (necrosis, tumor parenchyma) was calculated using the following formula [23,28]:

V_v_ (necrosis, tumor parenchyma) = ∑P(necrosis)/(κ·∑P(tumor parenchyma)

in which κ is the ratio between the number of grid points (P) used for targeting the necrosis and those used for the reference space. We planned a test grid in which 24 points were used for targeting the necrosis and six points for hitting the tumor parenchyma; hence, κ = 4.

For inferential statistics purposes, in queens with multiple synchronous malignant tumors of different histological types, a reference lesion was selected based on previously evaluated prognostic factors [29], namely the presence of lymphovascular invasion (primary criterion), higher EE histological grade (secondary criterion) or larger tumor diameter (tertiary criterion). Additionally, in cases with tumors of different biological behavior (benign vs. malignant), preference was given to the malignant neoplasms. Pearson’s Chi-squared test or Fisher’s exact test (when appropriate) were used to determine the significance of the association between necrosis and clinicopathological features. When applicable, data were presented as odds ratios (OR) with 95% confidence intervals (CI).

The interobserver agreement was calculated using weighted quadratic kappa (k). The following interpretation of k was considered: ≤0 = poor agreement, 0.01–0.20 = slight agreement, 0.21–0.40 = fair agreement, 0.41–0.60 = moderate agreement, 0.61–0.80 = substantial agreement and 0.81–0.99 = almost perfect agreement [30]. Spearman’s correlation coefficient was used to assess the relationship between the histological semi-quantitative assessment and the stereological quantification of necrosis. Kaplan–Meier method and log-rank tests were used to estimate and compare the OS, TSS and DFI. Statistical analysis was performed in IBM SPSS Statistics for Windows, version 28.0 (IBM Corp, Armonk, NY, USA). *p* values ≤ 0.05 (two-tailed) were considered statistically significant.

## 3. Results

One-hundred and fifty-four queens were diagnosed with mammary neoplasms, 97.4% (150/154) of which had at least one malignant mammary tumor, and the remaining 2.6% (4/154) had benign tumors. The majority of the animals were mixed breed (*n* = 147, 95.4%) and 4.5% (*n* = 7) were Persian. The mean age was 11.3 ± 3.1 years old (range, 3–20 years). At presentation, 61.0% (94/153) of the queens were intact. The age at ovariohysterectomy was known in 88.1% (52/59) of the spayed cats included in the study. Of these, 13.5% (7/52) were neutered before one year of age.

Of 150 queens with malignant neoplasms, 30.0% (*n* = 45) had multiple malignant tumors and 8.0% (*n* = 12) had concomitant benign tumors. The presence and level of necrosis was thus assessed in 231 mammary gland tumors of the 154 queens. Evidence of necrosis was noted in 33.3% (6/18) of the benign tumors and 87.8% (187/213) of the malignant tumors. Furthermore, necrosis was more extensive in the malignant tumors (mean V_v_ = 0.140 ± 0.147; range, 0.000–0.690) when compared to their benign counterparts (mean V_v_ = 0.005 ± 0.014; range, 0.000–0.060). Data on the presence of different types of necrosis are shown in Table 1.

### 3.1. Presence vs. Absence of Necrosis and Clinicopathological Features

Associations between the presence of different types of necrosis and clinicopathological features of the 150 reference malignant tumors are shown in Table 2. Every malignant neoplasm with necrosis had random foci of necrotic tissue. Age, mitotic score according to MGS, nuclear pleomorphism and lymph node status exhibited no significant associations with necrosis. Ischemic necrosis was mostly present in tumors with larger diameters (*p* = 0.009), ulceration (*p* = 0.012, OR = 2.510 [1.226–5.140]), clinical stage IV (*p* = 0.003), MGS and EE grade III (*p* = 0.020 and *p* = 0.013, respectively), higher mitotic count (EE) (*p* < 0.001), lymphovascular invasion (*p* = 0.002, OR = 3.000 [1.516–5.937]) and distant metastasis (*p* = 0.008, OR = 15.971 [2.075–122.946]). Random foci of necrosis were more frequently observed in tumors with larger diameters (*p* = 0.018), infiltrative growth (*p* = 0.006, OR = 5.857 [1.650–20.787]), multifocal-to-diffuse perilesional inflammatory infiltrate (*p* = 0.007, OR = 8.867 [1.822–43.158]), MGS grade II and III (*p* = 0.005), EE grade III (*p* = 0.040), higher mitotic count (EE) (*p* = 0.015) and abnormal nuclei (*p* = 0.013, OR = 4.652 [1.379–15.691]). Necrosis with a comedo pattern was more frequent in simple carcinomas (*p* = 0.018), with infiltrative growth (*p* = 0.046, OR = 3.222 [1.021–10.166]), EE grade III (*p* = 0.032), high mitotic count (EE) (*p* = 0.016) and abnormal nuclei (*p* = 0.013, OR = 2.883 [1.247–6.664]).

### 3.2. Level of Necrosis: Semi-Quantitative Assessment and Interobserver Agreement

The semi-quantitative assessment of necrosis by two different observers had an overall concordance percentage of 63.6% (147/231), with a resultant almost perfect agreement (k = 0.870, 95% CI = 0.831–0.908) (Table 3). An additional analysis of necrosis was performed considering a 50% cutoff (absence, <50% or ≥50%) and a 25% cutoff (absence, <25% or ≥25%). Upon statistical analysis, there was a concordance between the two observers of 84.0% (194/231) and 86.6% (200/231) when using the 50% and 25% cutoffs, respectively, reaching a substantial (50% cutoff) to almost perfect (25% cutoff) agreement (k_50%_ = 0.755, 95% CI = 0.682–0.829 and k_25%_ = 0.851, 95% CI = 0.750–0.878).

The presence of ≥25% of necrosis was significantly more frequent in larger, ulcerated tumors, with high MGS and EE histological grades, elevated mitotic scores (EE), multifocal-to-diffuse peritumoral inflammatory reactions and lymphovascular invasions, irrespective of the observer (*p* < 0.05). Furthermore, when considering the evaluation of Observer 2, a higher level of necrosis was also associated with tumors exhibiting an infiltrative behavior and presenting increased abnormal nuclei (*p* < 0.05). No significant associations were found between the extent of necrosis and the remaining clinicopathological features.

### 3.3. Level of Necrosis: Quantitative Stereological Assessment

In order to perform the Chi-squared test, a V_v_ cut-off of 0.130 was derived from the median of the 150 reference malignant tumors. A V_v_ value of ≥0.130 was more frequent in tumors with ulceration (*p* = 0.002, OR = 1.472 [1.472–5.975]), a higher EE and MGS grade (*p* = 0.026 and *p* = 0.003, respectively), high mitotic count (EE) (*p* < 0.001) and when lymphovascular invasion was detected (*p* = 0.006, OR = 2.538 [1.312–4.910]). No significant associations were observed between the V_v_ and other clinicopathological features.

**Figure 3:**
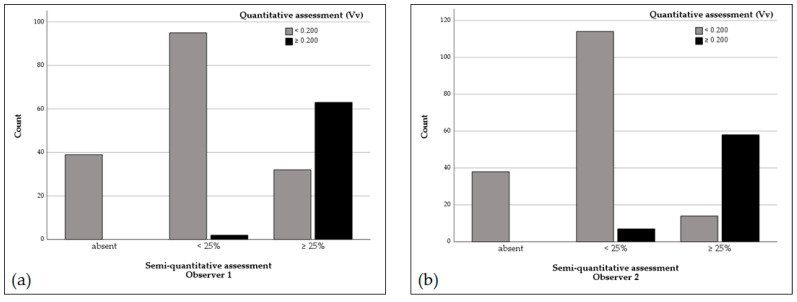
Bar graphs showing the distribution of cases across the categories of the semi-quantitative assessment of Observer 1 (**a**) and Observer 2 (**b**), considering a cutoff of 25%, each category separated according to the quantitative assessment.

Quantitative and semi-quantitative levels of necrosis were significantly correlated, regardless of the observer (*p* < 0.001; r_observer 1_ = 0.885, r_observer 2_ = 0.899). Cases with a V_v_ value > 0.200 were mostly classified as displaying over 25% of necrosis (Figure 3). In this study, the spatial limitation of the human eye was particularly noted in tumors with extensive central necrosis. During the semi-quantitative assessment of these tumors, the observers were under the illusion that necrosis occupied an area over 50% of the tumor, but this was not confirmed by stereology. In the objective, unbiased analysis with stereological methodology, the V_v_ of necrosis in those tumors was lower than expected and frequently less than 0.500.

### 3.4. Survival Analysis

Follow-up inclusion criteria regarding OS were met by 86 queens. At the end of the 2-year follow-up period, 59 (68.6%) cats were dead, 20 (23.3%) were still alive and 7 (8.1%) were lost to follow-up. The median OS was 9 months. Fifty queens died of tumor-related causes, with a median TSS of 8 months. No significant associations were observed regarding the influence of any of the necrosis variables on OS and TSS. However, the difference between the OS of the queens with and without ischemic central necrosis trended towards significance (*p* = 0.056), as queens with and without ischemic central necrosis showed a median OS of 6 and 13 months, respectively.

DFI data were available for 71 queens, 47 (66.2%) of which presented disease progression within two years post-surgery. The median DFI was 8 months. No significant associations were observed between DFIs and any of the necrosis variables. Notwithstanding, a tendency similar to that observed in the survival analysis was noted, as queens with ischemic central necrosis had a median DFI of 5 months, compared to a median of 10 months in those without.

## 4. Discussion

Herein, different types and patterns of tumoral necrosis, and their level determined by a semi-quantitative and a quantitative stereological approach were described in 231 FMTs. The interobserver agreement on the semi-quantitative estimation of necrosis and the correlation between semi-quantitative and stereological methods for quantifying necrosis were also determined for the first time in FMTs. Furthermore, the association of tumor necrosis with clinicopathological features and its prognostic value was evaluated.

Necrosis was observed in one-third of the benign tumors, often in a single, very discrete focus. The current histological classification of mammary tumors in domestic animals considers that random foci of necrosis are absent in benign tumors, but ischemic necrosis could be present [18]. Interestingly, in two-thirds of the benign tumors with necrosis, the necrotic foci were random, suggesting that this pattern of necrosis might be present in some benign neoplasms. Nonetheless, these findings might not be an accurate representation of these tumors, as the number of benign tumors in our cohort is very low. In this study, the majority of the malignant FMTs displayed some level of necrosis, which is in line with previous reports [11,12,14,16,17]. Ischemic central and random necrosis were more common in malignant neoplasms (51.2% and 78.4% of malignant tumors, respectively) than in benign neoplasms (11.1% and 22.2% of benign tumors, respectively), while necrosis with a comedo pattern was exclusively observed in malignant tumors. The high prevalence of necrosis in malignant FMTs is analogous to that described in human breast cancer, where up to 68.4% of the cases displayed areas of necrosis [31,32,33].

In recent years, there have been increased efforts to determine the agreement between veterinary pathologists on the application of grading and classification systems [21,34,35,36]. To the authors’ best knowledge, this is the first study addressing the interobserver agreement on necrosis evaluation in FMTs. When evaluating the proportion of necrosis, both observers performed a semi-quantitative visual assessment using a consensus definition of what would be considered necrotic tissue [2,9]. The definition of necrosis is often assumed to be straightforward by pathologists; however, in the particular case of necrosis within mammary gland lesions, there are some confounding factors. In fact, the presence of acidophilic secretion containing inflammatory cells, cell desquamation and exuberant inflammation with degenerated polymorphonuclear cells can challenge the identification of true necrotic areas. The preliminary phase of achieving consensus criteria of the parameter necrosis is considered essential in reproducibility studies and was applied in the present study [9]. In this case series, the interrater agreement on the semi-quantitative analysis of necrosis was substantial-to-almost-perfect. The few differences between the evaluation of the observers could be due to observer bias or might be justified by the presence of an extent of necrosis nearing a threshold, with one of the observers assigning it to the lower category, and the other observer opting for the upper category. Furthermore, it has been recognized that human visual perception is susceptible to errors, namely optical illusions [37,38,39]. Thus, caution must be taken when visually assessing areas/proportions. In an attempt to circumvent human subjectivity and the spatial limitation on the assessment of the three-dimensional magnitude of necrosis, a quantitative, unbiased stereological method was applied for the first time in FMTs. It is important to stress that both semi-quantitative and quantitative estimation of the level of necrosis in each tumor was performed in all of the slides resulting from a cross-section, without making any previous selection of a particular slide or areas within the slides. Despite this approach, it could not be excluded that the estimated level of necrosis does not accurately represent the level present in the whole tumor, particularly in those larger and more heterogenous. The multicentric nature of this study can present as a limitation, as it introduces variability in tissue-handling aspects which cannot be fully controlled, such as the time elapsed between tumor excision and placement of the tissue in formalin. Additionally, larger tumors take longer to fix. These differences can affect tissue preservation and give way to autolytic events, which can mimic necrosis [40]. In an attempt to diminish this potential effect, areas of obvious poor preservation (i.e., cells with pyknotic, hyperchromatic, often nude nuclei, which may or may not be detached from surrounding tissues, and with no signs of inflammation) were not considered necrotic tissue.

The unbiased, stereological estimation of necrosis in FMTs was well correlated with the level determined by the microscopic examination of the two observers. This is an interesting finding, considering that significant differences existed between the methods. For example, the stereological point-counting method used herein implies that only points superimposed to the necrotic debris should be counted. On the other hand, it was evident that among the necrotic debris there were, for instance, small blank spaces and scattered erythrocytes, which were considered integral components of the overall necrotic area in the semi-quantitative assessment. Another important point when assessing necrosis within tumors is the impact of sample processing, tissue retraction and the possibility of necrotic debris lost during tumor trimming and during automatic processing. While ischemic central necrosis was impacted by these factors and exhibited blank spaces caused by tissue retraction and/or loss of material, random necrosis (including comedo necrosis) frequently displayed negligible tissue retraction and material loss.

Stereology was relevant for supporting the use of the semi-quantitative method for accurately assessing necrosis in FMTs. Moreover, knowing that the level of necrosis could be relevant in terms of prognosis in FMTs, it is important to have a rapid and simple method for the duty pathologist assessing this feature.

Overall, in the present study, necrosis was particularly related to more aggressive clinicopathological features. The presence of ischemic necrosis as well as larger necrotic areas and higher V_v_ was positively associated with ulceration, thus corroborating previous works [15]. Our findings further support the conclusions drawn by Weijer and Hart (1983), indicating that tumors with extensive necrosis often display an infiltrative behavior and a chronic inflammatory reaction. Also, a recent approach revealed that total (intratumoral and stromal) cytotoxic CD8+ tumor-infiltrating lymphocytes were significantly associated with the presence of necrosis in FMC [14]. In human invasive breast cancer, the presence of necrosis has also been associated with a more prominent inflammatory response, as well as with larger tumors and a higher histological grade [33,41,42,43], echoing the results obtained in the present study.

Even though there were no statistically significant differences between groups in the survival analysis, queens bearing mammary tumors with necrosis, irrespective of its type and pattern, and those with extensive necrosis (≥25% and ≥0.130 V_v_) presented shorter survival times and DFIs. This was particularly evident when ischemic central necrosis and random necrosis were presented within the malignant tumors. Our results align with those of previous studies in FMC reporting poorer median OS times in queens with tumors with central necrosis of any type [12], or with a percentage of necrosis over 25% [13]. Seixas and colleagues (2011), similarly to our results, failed to observe significant differences in the DFI, although the interval was shorter in cases with extensive necrosis [19]. On the other hand, tumor necrosis was an independent survival prognostic factor in an earlier study on FMC considering the following categories: no necrosis, some necrosis, ≈30%, 60% and ≥90% [15]. Furthermore, a recent study categorizing necrosis as ≤25% or >25% found it to be a significant 1-year OS prognostic factor [11]. Upon analysis of the TSS, no differences were found between the groups of the evaluated necrosis variables, including when regarding ischemic central necrosis. One previous work by Dagher and colleagues (2019) assessed the prognostic value of central necrosis (presence vs. absence) on TSS, and the results were also not significant [12]. It is important to note that our study’s overall lack of significant results regarding the survival analysis might be justified by the small sample number obtained after stratification of the cohort.

Though a few clinicopathological characteristics have been recognized as reliable FMT prognostic markers [29], parameters such as the EE grading system are typically based on a set of established criteria, and emerging features might provide additional insights that are overlooked by these systems. The recent addition of the MGS grading scheme in the latest series of FMT classifications [18] highlights the ongoing efforts towards the improvement of histopathological diagnosis as new research emerges, showing that these frameworks are not set in stone. Although necrosis did not significantly impact prognosis, our findings suggest that this feature might bring additional insights on FMT behavior, and thus represents a potentially important feature to be taken into account during routine diagnostics, as it might help veterinary practitioners with decision making and tutor expectation management, and during the development of novel therapeutic approaches. In any case, one of the key contributions of our research is the application of a transparent methodology for the evaluation of tumor necrosis, ensuring that future studies can replicate and/or refine these methods, thus contributing to the broader goal of methodological rigor in veterinary oncology research [44].

## 5. Conclusions

Taken together, our data confirmed that tumoral necrosis, irrespective of its type and pattern, is more common and more extensive in malignant than in benign FMTs. Furthermore, necrosis was associated with clinicopathological features that have been linked to worse prognosis. These findings underline the relevance of necrosis in the pathogenesis and progression of mammary tumors in the feline species, as already documented in human breast cancer, thus further substantiating the FMT as a spontaneous model of human breast cancer. Furthermore, the cutoff of 25% of necrosis allowed a good agreement between observers, and a significantly positive relationship between the semi-quantitative and the quantitative method was achieved. Notwithstanding, and considering the huge steps of the digital pathology machine learning applications, we can anticipate that necrosis, as other morphologic features, could be more objectively quantified in whole slide imaging. For now, it might be useful from a clinical perspective to include the level of necrosis in the pathologist report of malignant FMTs, using a semi-quantitative approach.

## Figures and Tables

**Table 1:** Presence of necrosis in 231 feline mammary neoplasms.

	Benign Tumors (*n* = 18)		Malignant Tumors (*n* = 213)	
	*n*	%	*n*	%
Ischemic central necrosis				
Absent	16	88.9	104	48.8
Present	2	11.1	109	51.2
Random necrosis ^1^				
Absent	14	77.8	46	21.6
Present	4	22.2	167	78.4
Comedo necrosis				
Absent	18	100	138	64.8
Present	-	-	75	35.2

^1^ Includes comedo necrosis.

**Table 2:** Associations between presence of different types of necrosis and clinicopathological features of 150 malignant tumors (reference lesions).

	Ischemic Necrosis			Random Necrosis			Comedo Necrosis		
	Absent *n* (%)	Present *n* (%)	*p* Value	Absent *n* (%)	Present *n* (%)	*p* Value	Absent *n* (%)	Present *n* (%)	*p* Value
Age									
≤10 years	24 (40.0%)	36 (60.0%)		5 (8.3%)	55 (91.7%)		39 (65.0%)	21 (35.0%)	
>10 years	34 (40.4%)	53 (59.6%)	NS	7 (7.9%)	82 (92.1%)	NS	48 (53.9%)	41 (46.1%)	NS
Tumor growth									
Expansive	11 (55.0%)	9 (45.0%)		5 (25.0%)	15 (75.0%)		16 (80.0%)	4 (20.0%)	
Infiltrative	49 (37.7%)	81 (62.3%)	NS	7 (5.4%)	123 (94.6%)	**0.011**	72 (55.4%)	58 (44.6%)	**0.050**
Ulceration									
Absent	45 (47.9%)	49 (52.1%)		11 (11.7%)	83 (88.3%)		59 (62.8%)	35 (37.2%)	
Present	15 (26.8%)	41 (73.2%)	**0.015**	1 (1.8%)	55 (98.2%)	**0.032**	29 (51.8%)	27 (48.2%)	NS
Tumor size									
<2 cm	25 (59.5%)	18 (40.5%)		8 (19.0%)	34 (81.0%)		24 (57.1%)	18 (42.9%)	
2–3 cm	15 (37.5%)	25 (62.5%)		2 (5.0%)	38 (95.0%)		25 (62.5%)	15 (37.5%)	
>3 cm	18 (29.5%)	43 (70.5%)	**0.009**	2 (3.3%)	59 (96.7%)	**0.018**	34 (55.7%)	27 (44.3%)	NS
Clinical stage									
I	10 (50.0%)	10 (50.0%)		4 (20.0%)	16 (80.0%)		12 (60.0%)	8 (40.0%)	
II	10 (47.6%)	11 (52.4%)		2 (9.5%)	19 (90.5%)		13 (61.9%)	8 (38.1%)	
III	38 (44.2%)	48 (55.8%)		5 (5.8%)	81 (94.2%)		51 (59.3%)	35 (40.7%)	
IV	1 (5.0%)	19 (95.0%)	**0.003**	-	20 (100%)	NS	10 (50.0%)	10 (50.0%)	NS
Histological classification									
Simple carcinomas	29 (38.7%)	46 (61.3%)		5 (6.7%)	70 (93.3%)		39 (52.0%)	36 (48.0%)	
Ductal-associated carcinomas	4 (50.0%)	4 (50.0%)		3 (37.5%)	5 (62.5%)		8 (100%)	-	
Special-type carcinomas	2 (28.6%)	5 (71.4%)		-	7 (100%)		6 (85.7%)	1 (14.3%)	
Other ^a^	2 (50.0%)	2 (50.0%)	NS	-	4 (100%)	NS	2 (50.0%)	2 (50.0%)	**0.012**
Histological grade (MGS)									
I	15 (53.6%)	13 (46.4%)		7 (25.9%)	21 (75.0%)		21 (75.0%)	7 (25.0%)	
II	26 (47.3%)	29 (52.7%)		2 (3.6%)	53 (96.4%)		30 (54.5%)	25 (45.5%)	
III	17 (27.0%)	46 (73.0%)	**0.020**	3 (4.7%)	60 (95.2%)	**0.005**	35 (55.6%)	28 (44.4%)	NS
Histological grade (EE)									
I	7 (77.8%)	2 (22.2%)		2 (22.2%)	7 (77.8%)		8 (88.9%)	1 (11.1%)	
II	28 (44.4%)	35 (55.6%)		8 (12.7%)	55 (87.3%)		41 (65.1%)	22 (34.9%)	
III	23 (31.1%)	51 (68.9%)	**0.013**	2 (2.7%)	72 (97.3%)	**0.040**	37 (50.0%)	37 (50.0%)	**0.032**
EE mitotic score									
≤8	4 (80.0%)	1 (20.0%)		1 (20.0%)	4 (80.0%)		4 (80.0%)	1 (20.0%)	
9–17	17 (73.9%)	6 (26.1%)		5 (21.7%)	18 (78.3%)		19 (82.6%)	4 (17.4%)	
≥18	37 (31.4%)	81 (68.6%)	**<0.001**	6 (5.1%)	112 (94.9%)	**0.015**	63 (53.4%)	55 (46.6%)	**0.016**
Mills mitotic score									
≤62	56 (41.5%)	79 (58.5%)		12 (8.9%)	123 (91.1%)		78 (57.8%)	57 (42.2%)	
>62	2 (18.2%)	9 (81.8%)	NS	-	11 (100%)	NS	8 (72.7%)	3 (27.3%)	NS
Nuclear pleomorphism									
Mild	1 (33.3%)	2 (66.7%)		1 (33.3%)	2 (66.7%)		3 (100%)	-	
Moderate	32 (46.4%)	37 (53.6%)		8 (11.6%)	61 (88.4%)		41 (59.4%)	28 (40.6%)	
Marked	25 (33.8%)	49 (66.2%)	NS	3 (4.1%)	71 (95.9%)	NS	42 (56.8%)	32 (43.2%)	NS
Abnormal nuclear form									
≤5%	18 (47.4%)	21 (53.8%)		7 (18.4%)	32 (82.1%)		29 (76.3%)	10 (25.6%)	
>5%	40 (37.0%)	67 (62.6%)	NS	5 (4.6%)	102 (95.3%)	**0.016**	57 (52.8%)	50 (46.7%)	**0.024**
Perilesional inflammatory infiltrate									
Absent to focal	5 (62.5%)	3 (37.5%)		3 (37.5%)	5 (62.5%)		8 (100%)	-	
Multifocal to diffuse	55 (38.7%)	87 (62.3%)	NS	9 (6.3%)	133 (93.7%)	**0.018**	80 (56.3%)	62 (43.7%)	**0.021**
Lymphovascular invasion									
Absent	40 (52.6%)	36 (47.4%)		9 (11.8%)	67 (88.2%)		48 (63.2%)	28 (36.8%)	
Present	20 (27.0%)	54 (73.0%)	**0.002**	3 (4.1%)	71 (95.9%)	NS	40 (54.1%)	34 (45.9%)	NS
Lymph node metastasis									
Absent	18 (42.9%)	24 (57.1%)		2 (4.8%)	40 (95.2%)		23 (54.8%)	19 (45.2%)	
Present	21 (30.9%)	47 (69.1%)	NS	3 (4.4%)	65 (95.6%)	NS	41 (60.3%)	27 (39.7%)	NS
Distant metastasis									
Absent	58 (45.7%)	69 (54.3%)		11 (8.7%)	116 (91.3%)		76 (59.8%)	51 (40.2%)	
Present	1 (5.0%)	19 (95.0%)	**<0.001**	-	20 (100%)	NS	10 (50.0%)	10 (50.0%)	NS

NS—Not significant. ^a^ Includes sarcomas and carcinosarcomas. Bold values correspond to cases with significant statistical value (*p* ≤ 0.05).

**Table 3:** Crosstabulation of semi-quantitative assessment of necrosis in 231 feline mammary tumors performed by two independent observers.

Semi-Quantitative Assessment of Necrosis						
	Observer 1	Absent	<10%	10–24%	25–49%	≥50%
Observer 2						
Absent		38	0	0	0	0
<10%		0	61	14	2	1
10–24%		0	5	14	23	1
25–49%		1	0	3	16	33
≥50%		0	0	0	1	18

## Data Availability

The data presented in this study are available upon request from the corresponding author due to privacy or ethical reasons.

## References

[1] D’Arcy M.S. (2019). Cell Death: A Review of the Major Forms of Apoptosis, Necrosis and Autophagy. Cell Biol. Int..

[2] Miller M.A., Zachary J.F. (2017). General Pathology. Pathologic Basis of Veterinary Disease.

[3] Proskuryakov S.Y., Gabai V.L. (2010). Mechanisms of Tumor Cell Necrosis. Curr. Pharm. Des..

[4] Yee P.P., Li W. (2021). Tumor Necrosis: A Synergistic Consequence of Metabolic Stress and Inflammation. Bioessays.

[5] Coussens L.M., Werb Z. (2002). Inflammation and Cancer. Nature.

[6] Marioli-Sapsakou G.-K., Kourti M. (2021). Targeting Production of Reactive Oxygen Species as an Anticancer Strategy. Anticancer Res..

[7] Nakamura H., Takada K. (2021). Reactive Oxygen Species in Cancer: Current Findings and Future Directions. Cancer Sci..

[8] Avallone G., Rasotto R., Chambers J.K., Miller A.D., Behling-Kelly E., Monti P., Berlato D., Valenti P., Roccabianca P. (2021). Review of Histological Grading Systems in Veterinary Medicine. Vet. Pathol..

[9] Moore F.M., Williams, Bertram C.A., Donovan T.A., Klopfleisch R., Meuten D.J. (2021). Santos Tumor Necrosis Guideline, Version 1.1. Veterinary Cancer Guidelines and Protocols.

[10] Richards C.H., Mohammed Z., Qayyum T., Horgan P.G., McMillan D.C. (2011). The Prognostic Value of Histological Tumor Necrosis in Solid Organ Malignant Disease: A Systematic Review. Future Oncol..

[11] Chen B., Lin S.J.-H., Li W.-T., Chang H.-W., Pang V.F., Chu P.-Y., Lee C.-C., Nakayama H., Wu C.-H., Jeng C.-R. (2020). Expression of HIF-1α and VEGF in Feline Mammary Gland Carcinomas: Association with Pathological Characteristics and Clinical Outcomes. BMC Vet. Res..

[12] Dagher E., Abadie J., Loussouarn D., Campone M., Nguyen F. (2019). Feline Invasive Mammary Carcinomas: Prognostic Value of Histological Grading. Vet. Pathol..

[13] Mills S.W., Musil K.M., Davies J.L., Hendrick S., Duncan C., Jackson M.L., Kidney B., Philibert H., Wobeser B.K., Simko E. (2015). Prognostic Value of Histologic Grading for Feline Mammary Carcinoma: A Retrospective Survival Analysis. Vet. Pathol..

[14] Nascimento C., Gameiro A., Correia J., Ferreira J., Ferreira F. (2022). The Landscape of Tumor-Infiltrating Immune Cells in Feline Mammary Carcinoma: Pathological and Clinical Implications. Cells.

[15] Weijer K., Hart A.A. (1983). Prognostic Factors in Feline Mammary Carcinoma. J. Natl. Cancer Inst..

[16] Gameiro A., Nascimento C., Correia J., Ferreira F. (2021). HER2-Targeted Immunotherapy and Combined Protocols Showed Promising Antiproliferative Effects in Feline Mammary Carcinoma Cell-Based Models. Cancers.

[17] Gameiro A., Nascimento C., Urbano A.C., Correia J., Ferreira F. (2021). Serum and Tissue Expression Levels of Leptin and Leptin Receptor Are Putative Markers of Specific Feline Mammary Carcinoma Subtypes. Front. Vet. Sci..

[18] Zappulli V., Peña L., Rasotto R., Goldschmidt M.H., Gama A., Scruggs J.L., Kiupel M. (2019). Surgical Pathology of Tumors of Domestic Animals—Volume 2: Mammary Tumors.

[19] Seixas F., Palmeira C., Pires M.A., Bento M.J., Lopes C. (2011). Grade Is an Independent Prognostic Factor for Feline Mammary Carcinomas: A Clinicopathological and Survival Analysis. Vet. J..

[20] Meyer J.S., Alvarez C., Milikowski C., Olson N., Russo I., Russo J., Glass A., Zehnbauer B.A., Lister K., Parwaresch R. (2005). Breast Carcinoma Malignancy Grading by Bloom–Richardson System vs Proliferation Index: Reproducibility of Grade and Advantages of Proliferation Index. Mod. Pathol..

[21] Santos M., Correia-Gomes C., Santos A., de Matos A., Dias-Pereira P., Lopes C. (2015). Interobserver Reproducibility of Histological Grading of Canine Simple Mammary Carcinomas. J. Comp. Pathol..

[22] Santos M., Dias-Pereira P., Correia-Gomes C., Marcos R., de Matos A., Rocha E., Lopes C. (2017). Use of the Optical Disector in Canine Mammary Simple and Complex Carcinomas. APMIS.

[23] Santos M., Monteiro R.A.F., Rocha E. (2013). A Stereological Study of the Volume-Weighted Volume and of the Relative Volume of the Nucleus of Normal and Preneoplastic Hepatocytes in a Trout Model of Hepatocarcinogenesis. Exp. Toxicol. Pathol..

[24] Casanova M., Branco S., Veiga I.B., Barros A., Faísca P. (2021). Stereology in Grading and Prognosis of Canine Cutaneous Mast Cell Tumors. Vet. Pathol..

[25] Elston C.W., Ellis I.O. (1991). Pathological Prognostic Factors in Breast Cancer. I. The Value of Histological Grade in Breast Cancer: Experience from a Large Study with Long-Term Follow-Up. Histopathology.

[26] Rodrigues-Jesus J., Canadas-Sousa A., Oliveira P., Figueira A.C., Marrinhas C., Petrucci G.N., Gregório H., Tinoco F., Goulart A., Felga H. (2024). Distribution of Inflammatory Infiltrate in Feline Mammary Lesions: Relationship With Clinicopathological Features. Vet. Comp. Oncol..

[27] McNeill C.J., Sorenmo K.U., Shofer F.S., Gibeon L., Durham A.C., Barber L.G., Baez J.L., Overley B. (2009). Evaluation of Adjuvant Doxorubicin-Based Chemotherapy for the Treatment of Feline Mammary Carcinoma. J. Vet. Intern. Med..

[28] Freere R.H., Weibel E.R. (1967). Stereologic Techniques in Microscopy. J. R. Microsc. Soc..

[29] Zappulli V., Rasotto R., Caliari D., Mainenti M., Peña L., Goldschmidt M.H., Kiupel M. (2015). Prognostic Evaluation of Feline Mammary Carcinomas: A Review of the Literature. Vet. Pathol..

[30] Sim J., Wright C.C. (2005). The Kappa Statistic in Reliability Studies: Use, Interpretation, and Sample Size Requirements. Phys. Ther..

[31] Fisher E.R., Gregorio R.M., Fisher B., Redmond C., Vellios F., Sommers S.C. (1975). The Pathology of Invasive Breast Cancer. A Syllabus Derived from Findings of the National Surgical Adjuvant Breast Project (Protocol No. 4). Cancer.

[32] Fisher E.R., Palekar A.S., Gregorio R.M., Redmond C., Fisher B. (1978). Pathological Findings from the National Surgical Adjuvant Breast Project (Protocol No. 4). IV. Significance of Tumor Necrosis. Hum. Pathol..

[33] Leek R.D., Landers R.J., Harris A.L., Lewis C.E. (1999). Necrosis Correlates with High Vascular Density and Focal Macrophage Infiltration in Invasive Carcinoma of the Breast. Br. J. Cancer.

[34] Northrup N.C., Howerth E.W., Harmon B.G., Brown C.A., Carmicheal K.P., Garcia A.P., Latimer K.S., Munday J.S., Rakich P.M., Richey L.J. (2005). Variation among Pathologists in the Histologic Grading of Canine Cutaneous Mast Cell Tumors with Uniform Use of a Single Grading Reference. J. Vet. Diagn. Investig..

[35] Papparella S., Crescio M.I., Baldassarre V., Brunetti B., Burrai G.P., Cocumelli C., Grieco V., Iussich S., Maniscalco L., Mariotti F. (2022). Reproducibility and Feasibility of Classification and National Guidelines for Histological Diagnosis of Canine Mammary Gland Tumours: A Multi-Institutional Ring Study. Vet. Sci..

[36] Yap F.W., Rasotto R., Priestnall S.L., Parsons K.J., Stewart J. (2017). Intra- and Inter-Observer Agreement in Histological Assessment of Canine Soft Tissue Sarcoma. Vet. Comp. Oncol..

[37] Hamilton P.W., van Diest P.J., Williams R., Gallagher A.G. (2009). Do We See What We Think We See? The Complexities of Morphological Assessment. J. Pathol..

[38] Sabih D.-e., Sabih A., Sabih Q., Khan A.N. (2010). Image Perception and Interpretation of Abnormalities; Can We Believe Our Eyes? Can We Do Something about It?. Insights Imaging.

[39] Simundic A.-M., Nikolac N., Ivankovic V., Ferenec-Ruzic D., Magdic B., Kvaternik M., Topic E. (2009). Comparison of Visual vs. Automated Detection of Lipemic, Icteric and Hemolyzed Specimens: Can We Rely on a Human Eye?. Clin. Chem. Lab. Med..

[40] Taqi S.A., Sami S.A., Sami L.B., Zaki S.A. (2018). A Review of Artifacts in Histopathology. J. Oral Maxillofac. Pathol..

[41] Aaltomaa S., Lipponen P., Eskelinen M., Kosma V.M., Mari S., Alhava E., Syrjänen K. (1992). Histological Assessment of the Prognostic Factors in Female Breast Cancer. Oncology.

[42] Carlomagno C., Perrone F., Lauria R., de Laurentiis M., Gallo C., Morabito A., Pettinato G., Panico L., Bellelli T., Apicella A. (1995). Prognostic Significance of Necrosis, Elastosis, Fibrosis and Inflammatory Cell Reaction in Operable Breast Cancer. Oncology.

[43] Carter D., Pipkin R.D., Shepard R.H., Elkins R.C., Abbey H. (1978). Relationship of Necrosis and Tumor Border to Lymph Node Metastases and 10-Year Survival in Carcinoma of the Breast. Am. J. Surg. Pathol..

[44] Meuten D.J., Moore F.M., Donovan T.A., Bertram C.A., Klopfleisch R., Foster R.A., Smedley R.C., Dark M.J., Milovancev M., Stromberg P. (2021). International Guidelines for Veterinary Tumor Pathology: A Call to Action. Vet. Pathol..

